# Effects of Corrosion on Mechanical Properties of Welded Carbon Steel Pipe in District Heating Water

**DOI:** 10.3390/ma12223682

**Published:** 2019-11-08

**Authors:** Sang-Jin Ko, Jeong-Hun An, Yong-Sang Kim, Woo-Cheol Kim, Jung-Gu Kim

**Affiliations:** 1 School of Advanced Materials Science and Engineering, Sungkyunkwan University, 2066, Seobu-ro, Jangan-gu, Suwon 16419, Korea; tkdwls1315@naver.com (S.-J.K.); eiorid1727@gmail.com (J.-H.A.); skybyego@gmail.com (Y.-S.K.); 2 Technical Efficiency Research Team, Korea District Heating Corporation, 92 Gigok-ro, Yongin, Gyeonggi 06340, Korea; kwc7777@kdhc.co.kr

**Keywords:** carbon steel, welding, corrosion, mechanical properties

## Abstract

This study examined the effect of corrosion on mechanical properties of welded carbon steel pipe in district heating water. To evaluate the corrosion properties, potentiodynamic tests were conducted and a galvanostatic test was used to accelerate corrosion. Tensile tests and microstructure observations were performed to figure out the degradation of the corroded region, and stress intensity factors were calculated. As a result of the potentiodynamic tests, welded carbon steel pipe showed uniform corrosion and the total charge was calculated. Using the galvanostatic test, the current density at the equivalent aging time was applied to the specimens. The tensile tests showed that according to corrosion damages, mechanical properties were degraded due to corrosion. Through the microstructure observations and calculations of stress intensity factors, the corrosion of the welded carbon steel pipe induced the degradation of mechanical properties. The mode of fracture was changed from ductile to brittle fracture with increasing aging time.

## 1. Introduction

A district heating system is a utility energy service based on the supply of heat to remote customers from accessible heat sources [1]. A district heating system has several merits, such as being safe, convenient, and reducing costs without the need for additional equipment for heating in each individual building or household [2]. One of the most frequently used materials in district heating pipelines is a carbon steel pipe due to its availability and low cost [3,4,5,6,7,8].

Carbon steel pipe has advantages of availability and increased strength, but also has disadvantages of crack, fatigue, and corrosion problems in weld zone. Welding is a common method which is used for construction of long-distance district heating pipelines [9]. In the welding process of carbon steel pipe, the heat-affected zone (HAZ) is formed with several microstructures which are different from the base metal [10,11,12]. HAZ has different microstructures such as coarse grain ferrite, fine grain ferrite, and intercritical structures which are composed of freshly transformed martensite and ferrite. These different microstructures result in large variations in the strength values across the weld joints and cause electrochemical potential difference of individual parts in weldment. This induces corrosion among base metal-weld zone couples exposed to corrosive environments [13,14].

Due to the difference of corrosion rates among the base metal-weld zone, the deepest corroded point where stress concentration can occur is formed. Localized corrosion is regarded as the most hazardous type of damage since it requires less mass loss to cause pipe failures compared with uniform corrosion [15]. If stress concentration occurs at a point, the stress intensity factor (SIF) can be increased with the decrease of toughness and a brittle fracture can occur. Therefore, the failure of welded carbon steel pipes can be caused by this phenomenon. To investigate causes of the fractfure, the effect of corrosion on the mechanical properties has to be studied. There are many studies on the corrosion of steel reinforcement which leads to the degradation of the mechanical properties of the steel [16,17,18,19]. These conclude that the degradation of the mechanical properties was caused by the effective engineering stress that was increased by mass loss through corrosion. However, the materials in these studies were homogeneous, unlike welded carbon steel, and were confined to reinforcement bars. There is also a lack of research on the failure of welded carbon steel caused by corrosion.

In this study, welded carbon steel pipe (SPW 400) was used to evaluate the change of its mechanical properties according to corrosion accelerations performed at various quantities of electric charges. To investigate corrosion properties of the carbon steel pipe weldment, a potentiodynamic polarization test was carried out and the quantity of electric charge was calculated. For corrosion acceleration, the galvanostatic method based on results of the potentiodynamic tests was used. The mechanical properties according to the corrosion damages were evaluated by tensile tests. After that, microstructures of crack front were observed by optical microscope (OM) to investigate the change of crack initiation sites according to morphology. The fracture surfaces were observed by scanning electron microscope (SEM) to indicate the type of fractures. The stress analysis simulations and calculations of stress intensity factors were conducted to investigate the cause of failure.

## 2. Materials and Methods 

### 2.1. Specimen and Solution Preparation

**Figure 1:**
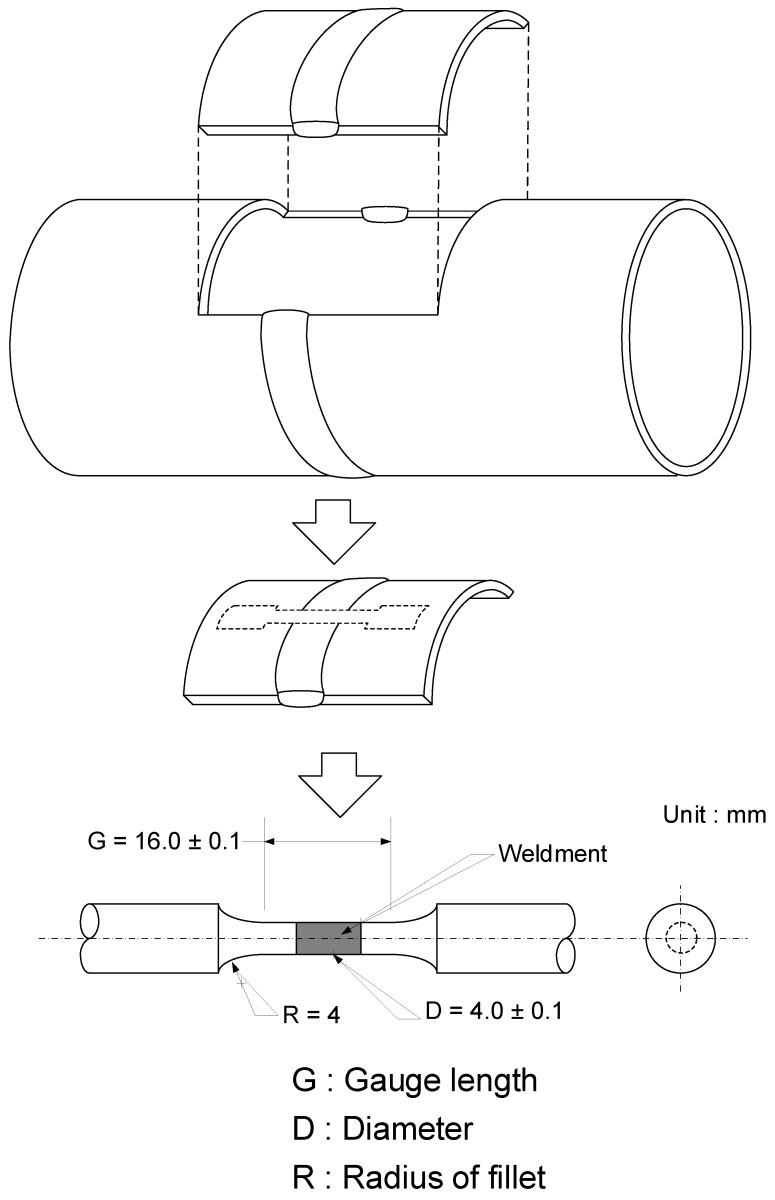
Schematic illustration of sectioning procedures to prepare tensile test specimen from welded pipes.

Carbon steel pipe for district heating system was used as the specimen and the chemical composition of the specimen is listed in Table 1. Pipes were welded by gas tungsten arc welding (GTAW) following specifications in Table 2. After welding, tensile test specimens were sectioned from welded pipes as shown in Figure 1.

For the electrochemical test, the surface of the specimen was polished with 600-grit silicon carbide (SiC) paper and rinsed in an ultrasonic bath with ethanol, and finally dried with N_2_ gas. Deaerated synthetic district heating water at 60 °C and a pH of 10.0 was prepared for the corrosion medium. The chemical composition of the synthetic district heating water is listed in Table 3.

### 2.2. Potentiodynamic Polarization Test

**Figure 2:**
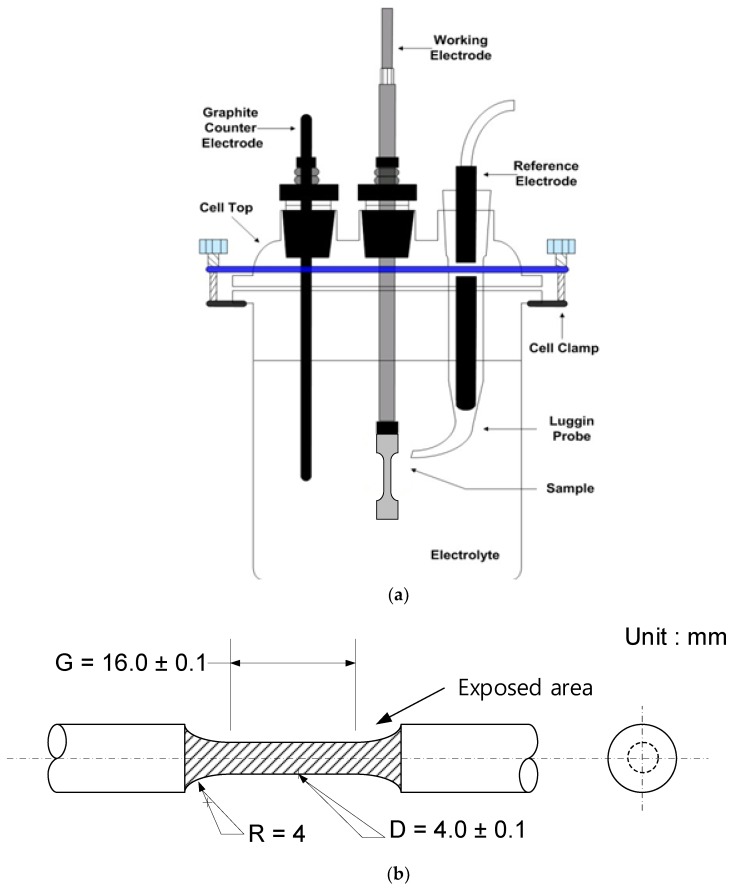
Schematic illustrations of (**a**) the three-electrode cell and (**b**) the exposed area of the specimen.

A potentiodynamic polarization test was carried out for evaluating corrosion properties by using a conventional three-electrode cell as shown in Figure 2a. The test specimen as the working electrode, a saturated calomel electrode (SCE) as the reference electrode, and graphite as the counter electrodes were used. The exposed area of the specimen was 3.9 cm^2^ from weld zone to base metal as shown in Figure 2b. The experiments were conducted in accordance with ASTM G5 [20] using a Model VSP-300 potentiostat (Biologic, Seyssinet-Pariset, France). An open circuit potential was established within 6 h before the electrochemical test. After that, a polarization test was performed at a scanning rate of 0.166 mV/s from −0.250 V_OCP_ to +1.6 V_SCE_.

### 2.3. Galvanostatic Method

The galvanostatic technique was used to accelerate corrosion by impressing anodic direct current [21]. The condition of the galvanostatic method from the potentiodynamic polarization test is listed in Table 4, where E_corr_ is the corrosion potential of the specimen, i_corr_ is the corrosion current density, and β_a_ and β_c_ are slopes of anodic and cathodic polarization curves in straight line section, respectively. By multiplying corrosion current density (i_corr_) by time (s) that materials were exposed to the corrosive environment, the charge amount (Q) induced to the specimens can be calculated. Corrosion damage according to the charge amount can be accelerated for the aging time of 0.4, 1, 2, and 4 years. Each aging time is matched to 811.7, 2435.2, 4058.7, and 8117.4 C charge amounts, respectively. The induced time of the galvanostatic method is calculated from the following equations.

(1) $$Q\;\left(C\right)=i_{corr}\left(A/cm^{2}\right)\times \mathrm{exposed}\;\mathrm{area}\;\left(cm^{2}\right)\times exposed\;time\;\left(s\right)$$

(2) $$\mathrm{Induced}\;\mathrm{time}\;\left(s\right)=Q\;\left(C\right)/\left[i_{\mathrm{app}}\;\left(A/{\mathrm{cm}}^{2})\times \mathrm{exposed}\;\mathrm{area}\;({\mathrm{cm}}^{2}\right)\right],$$

where i_corr_ is the corrosion current density of the specimen and i_app_ is the applied current density during the galvanostatic test. 

### 2.4. Tensile Test

To identify the effect of corrosion on the mechanical properties of a welded carbon steel pipe, a tensile test (KNR System, Yongin, Republic of Korea) was conducted for all five specimens including intact and corroded specimens. The weldment specimens for tensile testing were made from carbon steel pipe according to the ASTM E8 [22] as shown in Figure 1. The tensile tests were conducted at a strain rate of 10^−3^ s^−1^. The results were analyzed for the yield strength (YS), tensile strength (TS), and ductility (% total elongation).

### 2.5. Microstructure Observation

To investigate the effect of corrosion on the welded carbon steel pipe, microstructure observation was conducted. The specimens were polished with SiC paper to 2000 grit, and then polished using diamond suspensions down to 1 μm. After that, the specimens were etched with a 2% nital etchant for 10 s. Photomicrographs of weldment areas were taken using a Leica DM2700 M optical microscope (OM, Wetzlar, Germany).

### 2.6. FEM Simulation

**Figure 3:**
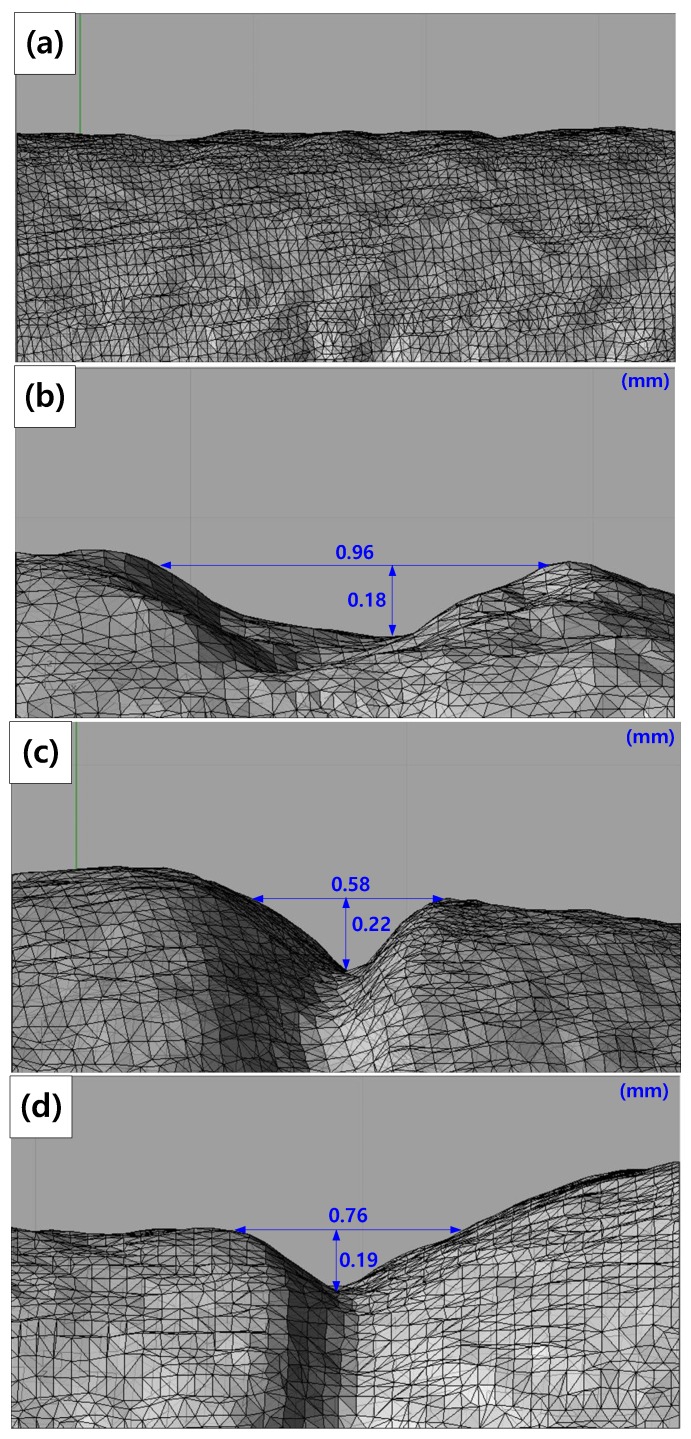
3D scan images of specimens after using the galvanostatic method: (**a**) 3% reduction, (**b**) 10% reduction, (**c**) 20% reduction, and (**d**) 50% reduction ratios.

**Figure 4:**
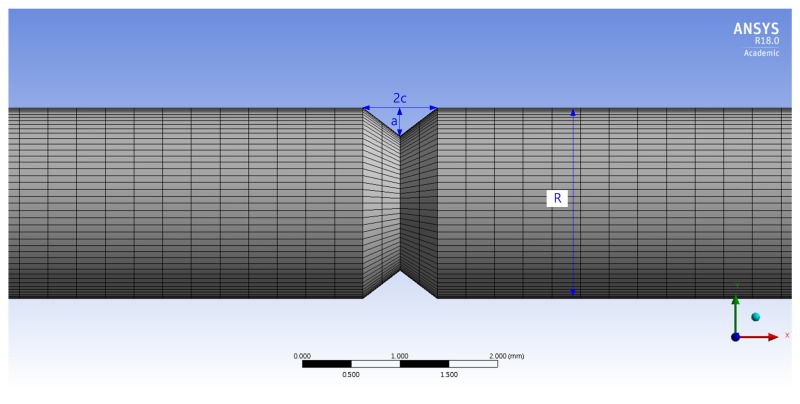
Meshed 3D model applied to computer simulation.

To investigate the maximum stress distribution of tensile specimens that were corroded according to the charge amounts, a numerical simulation was conducted using the finite element method (FEM, ANSYS 18.0, ANSYS, Canonsburg, PA, USA). The deepest corroded points were modeled by the 3D scan data of corroded specimens in Figure 3. In these 3D scan images, crack depth (a) and crack width (2c) were measured at the deepest corroded point. In Table 5, a, 2c, and diameter (R) measured from each specimen are listed. The corroded tensile specimen was modeled by the observed pit shape in Table 5. The mesh size of the specimens was fixed at 0.3 mm using the multizone method as shown in Figure 4.

**Figure 5:**
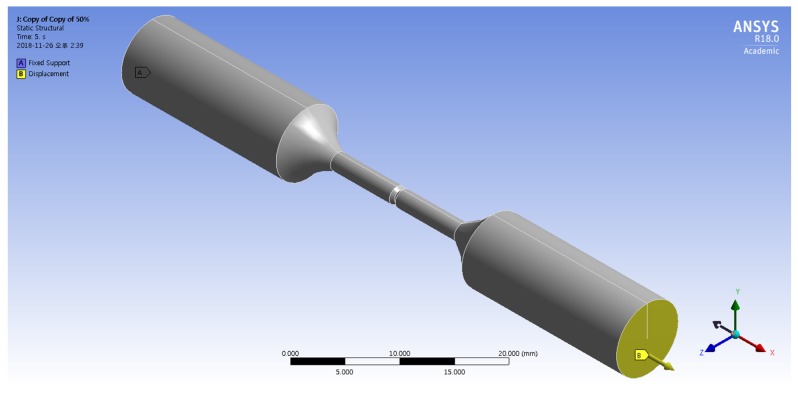
Boundary conditions for finite element method (FEM) simulation of a corroded tensile specimen.

Figure 5 shows the boundary conditions for the FEM simulation of the corroded tensile specimen. The fixed support was applied on the surface at the end of the tensile specimen. The displacement in the x-direction with 0.016 mm/s was applied on the surface at the opposite side of the tensile specimen.

### 2.7. Instrumented Indentation Test

**Figure 6:**
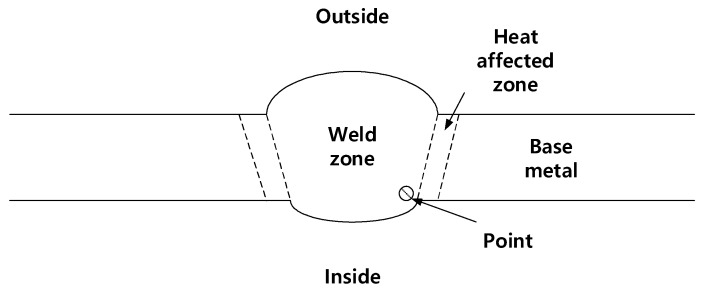
Schematic illustration of welded pipes for instrumented indentation technique.

To measure the fracture toughness value in mode 1 (K_1C_) of weldment where cracks occurred, the instrumented indentation technique was used. The indenter used for this test was a spherical indenter with a 0.5 mm diameter. FRONTICS’s instrumented indentation technique (FRONTICS, Seoul, Republic of Korea) was performed on the welded joint as shown in Figure 6. The maximum indentation depth was 150 μm and the rate of indentation loading/unloading was 30 μm/min. The point in Figure 6 represents the position where the indenter contacted the specimen surface.

## 3. Results

### 3.1. Potentiodynamic Polarization Test

**Figure 7:**
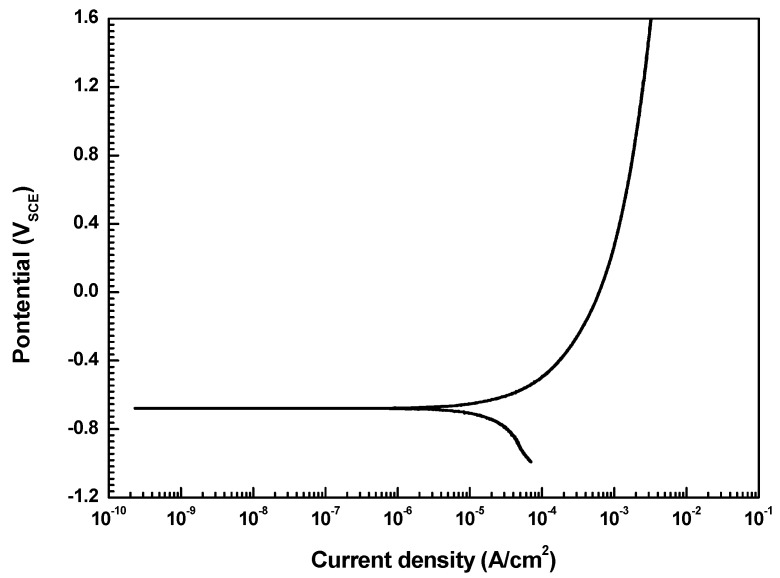
Potentiodynamic polarization curve of welded carbon steel in a deaerated synthetic district heating water at 60 °C and a pH of 10.0.

To investigate the corrosion behavior of welded carbon steel pipe, a potentiodynamic polarization test was conducted. The corrosion current density (i_corr_) was determined by the Tafel extrapolation method, and the corrosion rate can be inferred from the i_corr_ based on Faraday’s law [23]:

(3) $$\mathrm{Corrosion}\;\mathrm{rate}\;\left(\frac{\mathrm{mm}}{\mathrm{yr}}\right)=\frac{\left(0.00327\times i_{\mathrm{corr}}\times E.W.\right)}{D},$$

where 0.00327 is the metric and time conversion factor, i_corr_ is the corrosion current density (μA/cm^2^), E.W. is the equivalent weight (g), and D is the density (g/cm^3^). Figure 7 shows the results of the potentiodynamic polarization test. The corrosion current density measured by the Tafel extrapolation method is 16 μA/cm^2^. Table 6 lists the conditions of the accelerated test based on the results of the polarization test. As a result of the test, uniform corrosion occurred in the specimen.

### 3.2. Corrosion Acceleration by Galvanostatic Test Method

**Figure 8:**
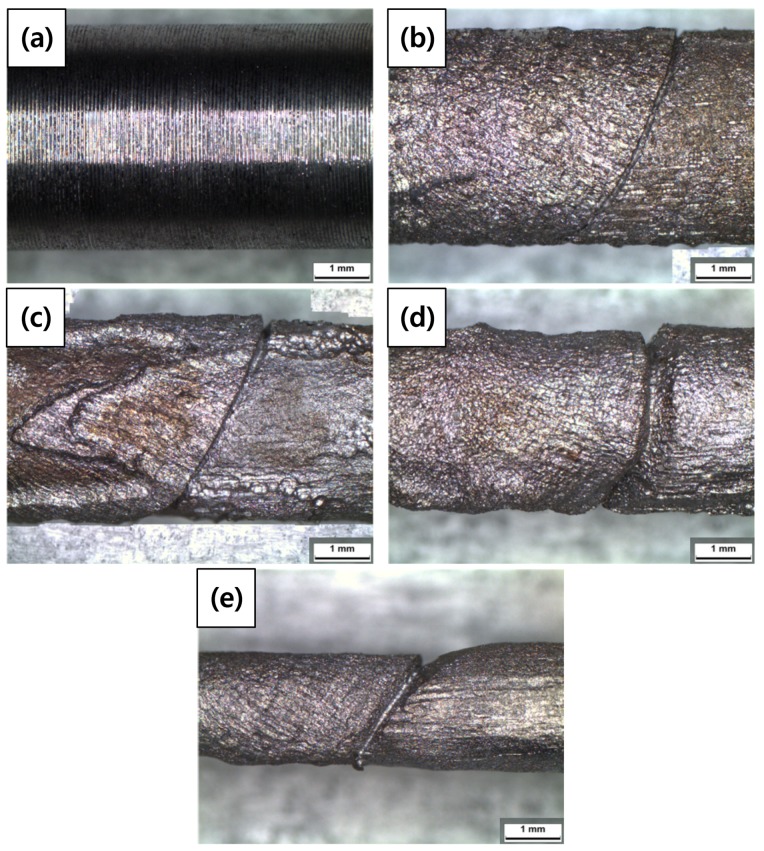
Optical microscope (OM) images of the welded carbon steel specimens after a galvanostatic test according to charge amounts: (**a**) 0%, (**b**) 3%, (**c**) 10%, (**d**) 20%, and (**e**) 50% reduction ratios.

Figure 8 and Table 7 show the images of the welded carbon steel specimen and the diameter reductions of the specimens after the galvanostatic test, respectively. Corrosion damage according to the acceleration time is classified into the diameter reduction ratio. As the acceleration time increased, the diameter reduction ratio increased. A crack-like scratch was observed between the weldment and base metal. 

### 3.3. Tensile Test

**Figure 9:**
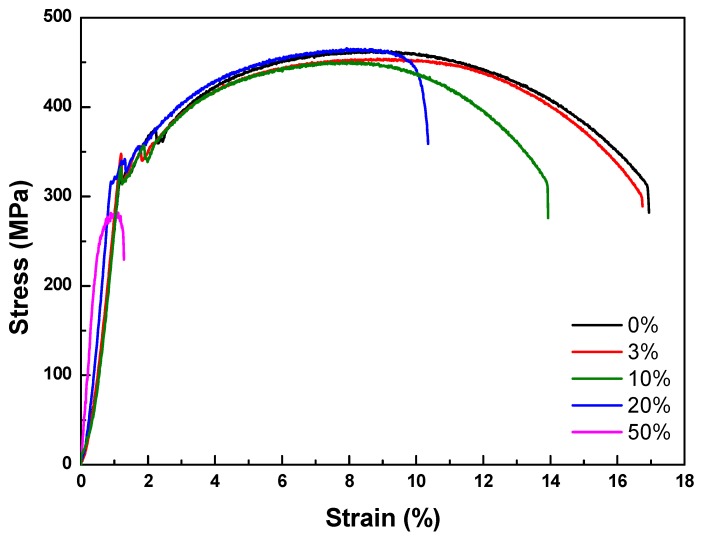
Stress–strain curves of accelerated corrosion specimens.

**Figure 10:**
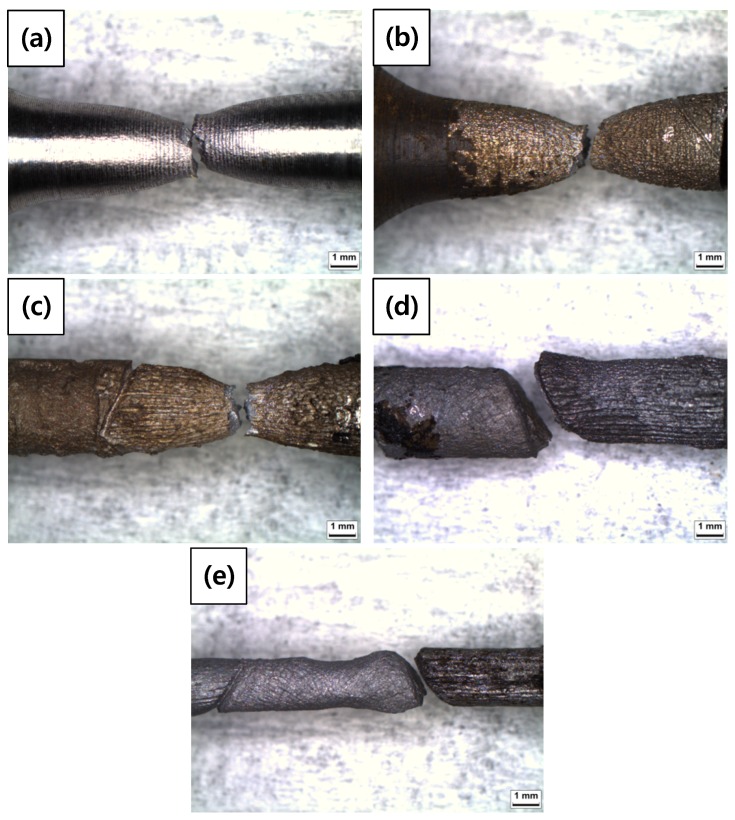
OM images of fracture morphologies of the corroded tensile specimens after tensile tests: (**a**) 0%, (**b**) 3%, (**c**) 10%, (**d**) 20%, and (**e**) 50% reduction ratios.

**Figure 11:**
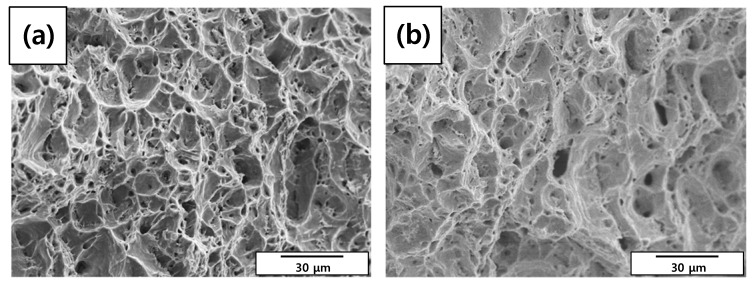

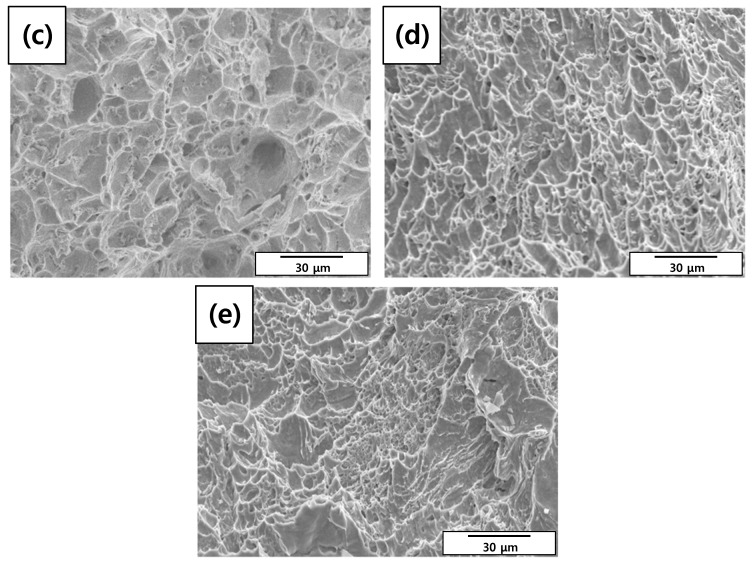
Scanning electron microscope (SEM) images of fracture surface morphologies of the corroded tensile specimens after tensile tests: (**a**) 0%, (**b**) 3%, (**c**) 10%, (**d**) 20%, and (**e**) 50% reduction ratios.

A tensile test was conducted to measure the mechanical properties of each specimen. As shown in Table 8 and Figure 9, elongation decreased as the diameter reduction ratio increased. Yield stress and tensile stress decreased remarkably under the 50% reduction condition. These results were consistent with the magnified images of fracture morphology and surface after the tensile tests. As shown in Figure 10, the fracture morphologies of the 20% and 50% reduction specimens were different compared to the others. They showed brittle fracture characteristics. Compared to the cup and cone type, their fracture shapes were flat with diagonal direction. As shown in Figure 11, a shallow dimple in the fracture surface indicated that a brittle fracture occurred rather than a ductile fracture [24]. In other specimens, ductile fracture surfaces with dimples were observed. The corrosion damages according to the charge amounts lead to the degradation of mechanical properties in the welded specimen. 

### 3.4. Microstructural Analysis

**Figure 12:**
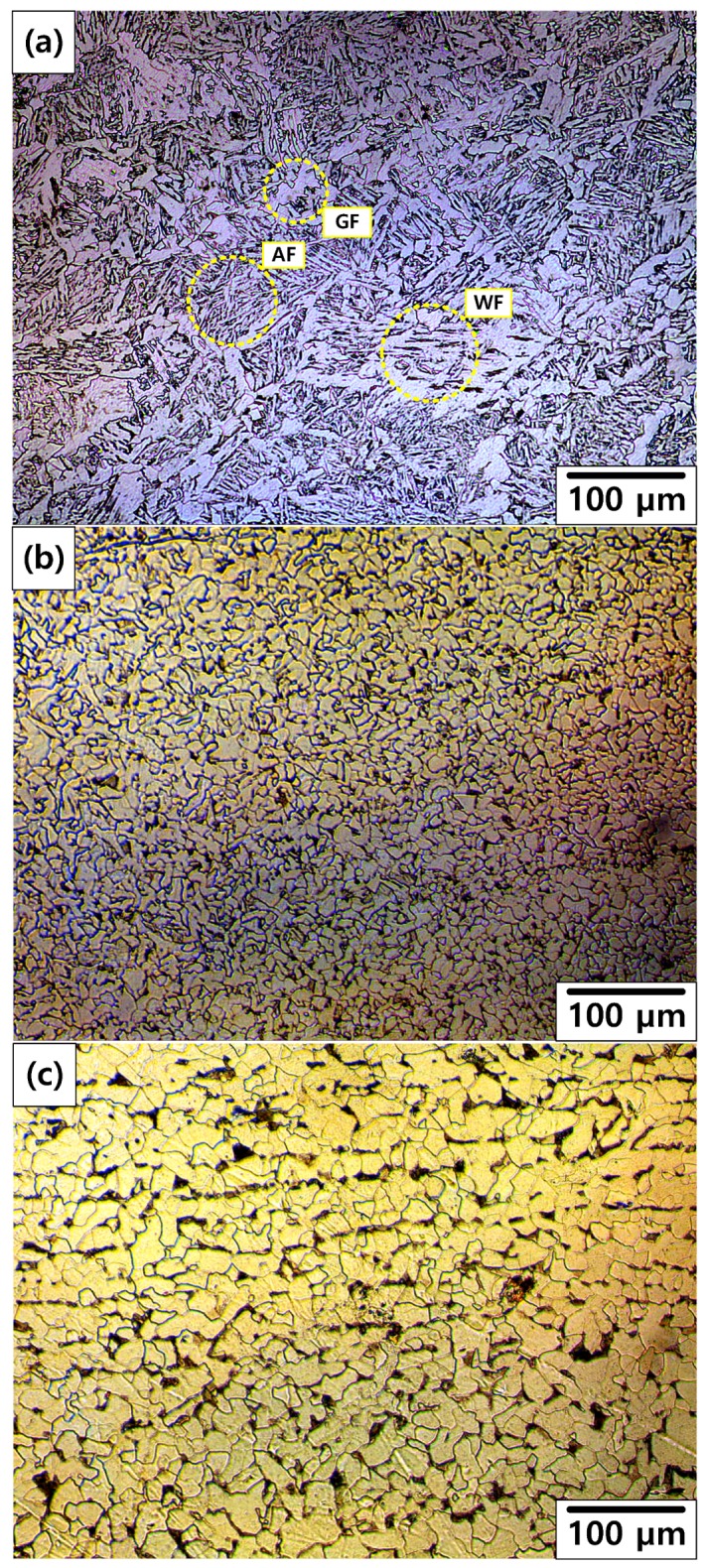
OM of welded carbon steel specimens from the weld zone to base metal: (**a**) Weld zone, (**b**) heat affected zone, and (**c**) base metal.

Figure 12 shows the OM images of the cross section of the welded carbon steel specimen from the weld zone to base metal. A typical microstructure of the weld zone of carbon steel pipe deposited by a GTAW process is shown in Figure 12a [25]. The structure in the as-received specimen is composed of acicular ferrite (AF), Widmanstatten ferrite (WF), and grain-boundary ferrite (GF) nucleated along the austenite grain boundary. The microstructure of HAZ and base metal (BM) showed an equiaxed ferrite (EF) microstructure with small pearlite (P) colonies, as shown in Figure 12b,c. However, grain size of HAZ was smaller than that of BM. As grain size decreased, corrosion rate increased in the case of the EF microstructure [26]. Furthermore, to prevent the large cathode/small anode effect, the weld zone was designed to have a higher potential than those of HAZ and BM. As a result of the galvanic corrosion, the corrosion rate of the weld zone was lower than those of HAZ and BM. Therefore, the corrosion rate of HAZ had the highest value in this work. These results are consistent with the following corrosion acceleration results.

**Figure 13:**
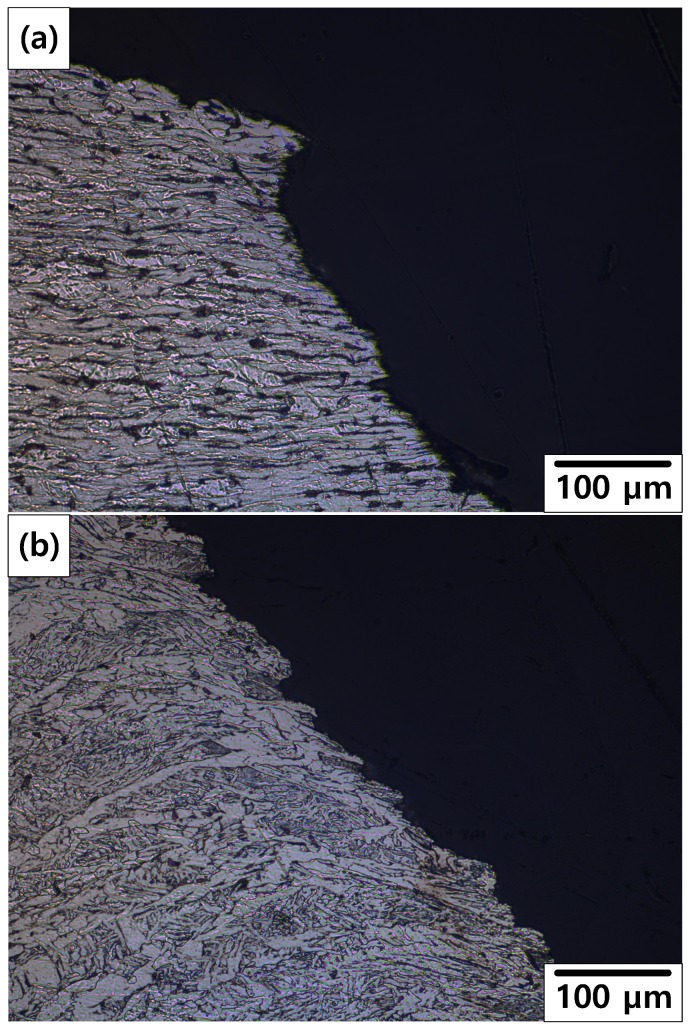
OM images of the cross section of the fracture fronts: (**a**) 10% reduction specimen and (**b**) 50% reduction specimen.

Figure 13 shows the OM images of the cross section of the fracture fronts. As shown in Figure 13a, the stretched EF microstructure was observed on the 10% reduction specimen. As shown in Figure 13b, typical microstructure of the weld zone was observed on the 50% reduction specimen. It is suspected that the position of the fracture changed from BM or HAZ to weld metal as reduction increased. This indicates that a crack can occur more easily near the weldment after more severe corrosion.

### 3.5. Stress Analysis and Stress Intensity Factor

**Figure 14:**
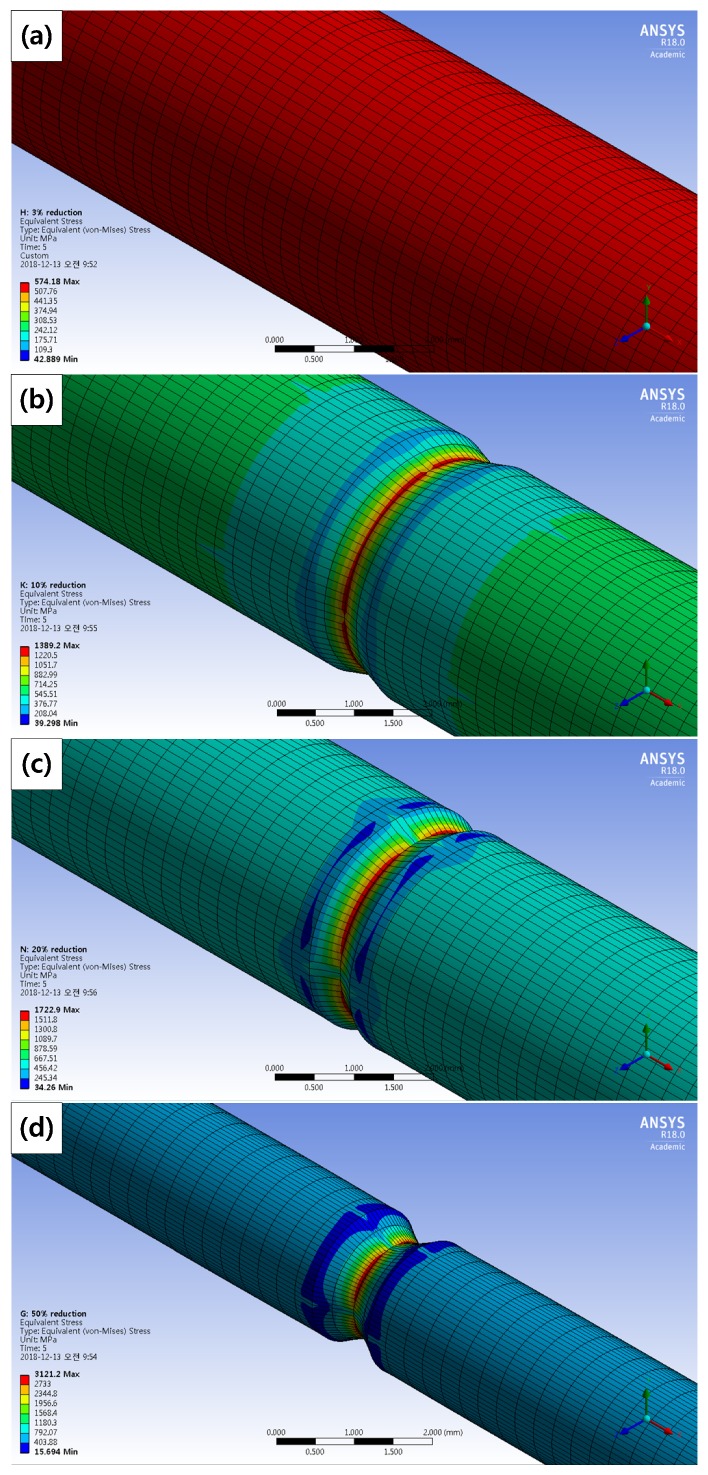
Maximum stress distribution of the corroded tensile specimens according to the diameter reduction by computer simulation: (**a**) 3%, (**b**) 10%, (**c**) 20%, and (**d**) 50% diameter reduction specimens.

Figure 14 shows the maximum stress distribution of the corroded tensile specimens under the condition (strain rate = 10^−3^ s^−1^) by the FEM simulation. The maximum stress according to the diameter reduction ratio from Figure 3 is listed in Table 9. As diameter reduction ratios increased, the maximum stress increased. Maximum stress appeared at the deepest corroded points in the 10%, 20%, and 50% reduction specimens. It is thought that a ductile fracture occurred because the maximum stress exceeded the yield stress (463.40 MPa) in 3% and 10% reduction specimens. Although stress concentration occurred in the 10% reduction specimen, its cross-section of fracture showed a ductile fracture. Brittle fracture occurred in 20% and 50% reduction specimens. Maximum stress distribution is not sufficient to investigate the cause of fracture-type change. 

To investigate the cause of the fracture type change, the stress intensity factor (SIF, K) was calculated. The stress intensity factor is generally applied to determine the risk of brittle fracture in a crack on the surface. SIF is based on the theory of fracture mechanics and has been employed to quantify the asymptotic stress distribution close to a locally corroded region [27]. For the specific geometry of a structure, SIF is affected not only by the applied stresses, but also by the overall geometry of the structure and crack. Mode I stress intensity factor (K_I_) for a crack or pitting on a pipe surface can be expressed as follows [28,29]:

(4) $$K_{I}\;=\;Y\sigma_{0}\sqrt{\pi a\;},$$

where *σ*_0_ is the applied stress, *Y* is a function that depends on both crack and specimen sizes and geometries, and *a* is the depth of the crack (corroded surface). In this work, the maximum stress was used as the value of Yσ_0_ for calculation.

**Figure 15:**
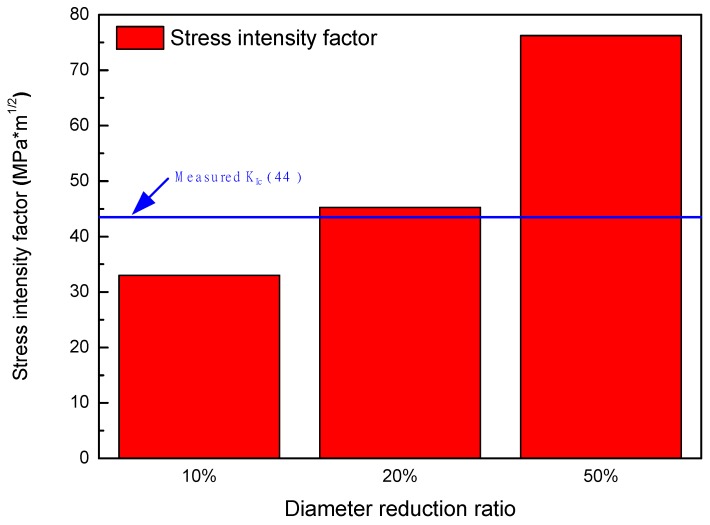
Stress intensity factors calculated for cases of the corroded tensile specimens according to diameter reduction ratios.

SIF values calculated by using the results of FE simulation for the specimens according to diameter reduction ratios are listed in Table 9 and shown in Figure 15. Under the 10% reduction condition, K_I_ (33.03 MPa m^1/2^) was lower than the measured K_Ic_ (44 MPa m^1/2^), which means that brittle fracture at the deepest corroded point could not occur. However, K_I_ values in the 20% reduction (45.28 MPa m^1/2^) and 50% reduction (76.23 MPa m^1/2^) were higher than the K_Ic_ value. This indicated that geometric changes due to corrosion increased the SIF and induced brittle fracture of the welded joint. 

## 4. Conclusions

In this study, the effect of corrosion on mechanical properties of welded carbon steel pipe was investigated using electrochemical and mechanical analyses. Using the galvanostatic method, the deepest corroded point between weld zone and base metal region was observed on the specimens. This suggests that corrosion was concentrated due to the dissimilarity of the microstructure. The results of the tensile test show that 3% and 10% diameter reduction specimens had no considerable degradation. The ductile fracture appeared regardless of the deepest corroded point. However, 20% reduction specimens showed a great decrease of elongation and 50% diameter reduction specimens showed remarkable decrease of elongation and strength values. In addition, 20% and 50% diameter reduction specimens showed brittle fracture at the deepest corroded point. As the diameter reduction increased, the maximum principal stress evaluated by FEM method increased. Unlike that of the 10% reduction specimen, stress intensity factors of 20% and 50% diameter reduction specimens were higher than the fracture toughness of weldment, the latter of which was measured by an instrumented indentation technique. Furthermore, in OM observation, it was determined that the position of the fracture changed from BM or HAZ to weld metal with the increase of diameter reduction. This indicates that a crack can occur more easily near the weldment after more severe corrosion. Therefore, corrosion leads to degradation of the mechanical properties of welded carbon steel pipe by the formation of stress concentration geometry. It increases the stress intensity factor of the specimen and the risk of brittle fracture in a district heating water environment.

## Figures and Tables

**Table 1:** Chemical composition of carbon steel pipe used in the district heating system.

Elements	C	Mn	P	S	Fe
**Composition** **(wt.%)**	0.25	1.00	0.040	0.040	Bal.

**Table 2:** Welding procedure specifications.

Welding Process	Gas Tungsten Arc Welding (GTAW)
Joint design	Single-V joint with a 65 ± 5 degree included angle and a 1.6 ± 0.8 mm root face
Number of passes	1–2 or higher
Electrode	ER70S-G (2.4 or 3.2 mm diameter, Thoriated, TGC-50)
Voltage	11–15 V or 22–28 V
Current	80–130 A or 100–170 A
Polarity	Direct current straight polarity (DCSP)
Travel speed	5–10 cm/min or 6–16 cm/min
Welding atmosphere	Argon, 7–17 L/min

**Table 3:** Chemical composition of the synthetic district heating water (mg/L).

Elements	NaCl	Mg(OH)_2_	CaCO_3_	NH_4_OH
Composition	15.01	0.48	2.65	10.28

**Table 4:** Results of a potentiodynamic polarization test for the weldment specimen in a deaerated synthetic district heating water at 60 °C and a pH of 10.0.

Specimens	E_corr_ (mV_SCE_)	i_corr_ (μA/cm^2^)	β_a_ (V/Decade)	β_c_ (V/decade)	Corrosion Rate (mm/Year)
As-received	−678.5	16.4	0.021	0.038	0.2

**Table 5:** Crack depth (a), crack width (2c), diameter (R), and measured diameter reduction ratio of each specimen.

Specimens (% Diameter Reduction)	a (mm)	2c (mm)	R (mm)
0%	–	–	3.95
3%	–	–	3.87
10%	0.18	0.96	3.53
20%	0.22	0.58	3.18
50%	0.19	0.76	1.96

**Table 6:** Conditions of the galvanostatic test (S = 3.9 cm^2^).

Charge Amounts	i_corr_	Equivalent Aging Time (Year)	i_app_	Test Time (h)
0	16 uA/cm^2^	0	1 mA/cm^2^	0
811.7		0.4		57.8
2435.2		1		173.5
4058.7		2		289.1
8117.4		4		578.2

**Table 7:** Measured diameter reduction of the corroded tensile specimens.

Charge Amounts	Diameter Reduction Ratio (%)	Diameter (mm)	Diameter Reduction (mm)
–	0	3.95	–
811.7	3	3.87	0.08
2435.2	10	3.53	0.42
4058.7	20	3.18	0.77
8117.4	50	1.96	1.99

**Table 8:** Mechanical properties of the corroded tensile specimens.

Specimens	Mechanical Properties		
	Yield Stress (MPa)	Tensile Stress (MPa)	Elongation (%)
0% reduction	374.54	463.40	16.94
3% reduction	347.74	454.13	16.75
10% reduction	332.62	450.62	13.93
20% reduction	336.27	465.62	10.36
50% reduction	208.09	282.12	1.28

**Table 9:** Maximum stress from FEM simulations, crack depth (a), and stress intensity factors of the corroded tensile specimens.

Specimens	Maximum Stress (MPa)	a (mm)	Stress Intensity Factor $\left(\mathrm{MPa}\sqrt{m}\right)$
0% reduction	–	–	–
3% reduction	578.06	–	–
10% reduction	1389.2	0.18	33.03
20% reduction	1722.9	0.22	45.28
50% reduction	3121.2	0.19	76.23
